# Holocene and contemporary marine dinoflagellate community patterns predict expansion of generalist dinoflagellate blooms in warming oceans

**DOI:** 10.1093/ismejo/wraf095

**Published:** 2025-05-14

**Authors:** Lemian Liu, Siqi Zhu, Yifan Gu, Shuqin Tao, Bernd Krock, Changyou Wang, Xinguo Shi, Qi Yan, Xiusong Pan, Jianfeng Chen, Senjie Lin, Zhaohe Luo

**Affiliations:** College of Biological Science and Engineering, Fuzhou University, Wulongjiang North Road, Fuzhou, Fujian, 350108, China; College of Biological Science and Engineering, Fuzhou University, Wulongjiang North Road, Fuzhou, Fujian, 350108, China; Department of Marine Sciences, University of Connecticut, 1084 Shennecossett Road, Groton, CT, 06340, United States; Third Institute of Oceanography, Ministry of Natural Resources, 178 Daxue Road, Xiamen, Fujian, 361005, China; Alfred Wegener Institut-Helmloltz Zentrum für Polar- und Meeresforschung, Am Handelshafen 12, Bremerhaven, D-27570, Germany; School of Marine Sciences, Nanjing University of Information Science and Technology, 219 Ningliu Road, Nanjing, Jiangsu, 210044, China; College of Biological Science and Engineering, Fuzhou University, Wulongjiang North Road, Fuzhou, Fujian, 350108, China; College of Biological Science and Engineering, Fuzhou University, Wulongjiang North Road, Fuzhou, Fujian, 350108, China; College of Biological Science and Engineering, Fuzhou University, Wulongjiang North Road, Fuzhou, Fujian, 350108, China; College of Biological Science and Engineering, Fuzhou University, Wulongjiang North Road, Fuzhou, Fujian, 350108, China; Department of Marine Sciences, University of Connecticut, 1084 Shennecossett Road, Groton, CT, 06340, United States; Third Institute of Oceanography, Ministry of Natural Resources, 178 Daxue Road, Xiamen, Fujian, 361005, China

**Keywords:** dinoflagellate, sedimentary ancient DNA, harmful algal bloom, climate warming, human activity, South China Sea

## Abstract

Existing data and models suggest increasing prominence of dinoflagellates and their blooms in future warmer ocean but supporting long-term data are sparse. Here, we used 18S rRNA gene sequencing to investigate sedimentary ancient dinoflagellate communities in northern South China Sea and compared them with contemporary dinoflagellate data from global oceans (*TARA Oceans* data) and 40 years of dinoflagellate bloom records in China. We found a continuous warming (by ~4.3°C in mean annual sea surface temperature) from 12 to 4.3 kiloyears before present (kyr BP), which caused an initial increase in the relative abundance and diversity of dinoflagellates, followed by a decrease reaching the lowest value, probably due to thermal stress. However, dinoflagellates flourished again after 4.3 kyr BP, coinciding with a rapid increase in human activities. Further analyses indicated that warming and environmental changes during the Holocene favored dinoflagellate generalists over specialists. These generalists have also been abundant throughout contemporary low- and mid-latitude regions, whereas specialists were more abundant at higher latitudes. The predominant generalist genera *Noctiluca*, *Gymnodinium*, and *Prorocentrum* in core sediment corresponded to taxa responsible for most dinoflagellate blooms in the contemporary China Seas over the past 40 years. The success of generalists during warmer periods suggests that dinoflagellate blooms are likely to expand geographically rather than simply shift toward high latitudes under global warming. Moreover, the homogenization of dinoflagellate communities resulting from generalist expansion may significantly reduce the complexity of marine plankton interactions and compromise ecosystem services under global warming.

## Introduction

Dinoflagellates are important components of the marine plankton and a key base of marine food webs, but can sometimes cause harmful effects on marine ecosystems when they form blooms [1, 2]. Harmful dinoflagellate blooms (HDBs) can have devastating impacts on marine ecosystems and coastal economies as many dinoflagellates produce toxic substances, which cause illness or death in fish, shellfish, marine mammals, and birds [3]. Other dinoflagellates and bloom-forming protists are nontoxic but their massive blooms can cause physical damage, such as clogging the gills of fish and invertebrates, or lead to oxygen depletion in the water as they decay.

Climate warming has profound effects on dinoflagellate communities and has garnered significant research attention in recent decades. Most studies in middle and high latitude regions suggest that global warming has aggravated dinoflagellate blooms [4–6]. Warmer water temperatures can intensify dinoflagellate blooms if the temperature remains below the optimal range for maximal growth. However, when warming exceeds this optimal range, bloom intensities may plateau or even decrease [7]. Consequently, HDBs may migrate poleward with rising temperatures [4, 5].

Despite the general hypothesis that warming drives poleward shift of organisms’ distribution, different dinoflagellates respond differently to warming due to variations in niche breadth. Some species are generalists, able to thrive in a wide range of conditions, whereas others are specialists which adapt to specific habitats [8, 9]. For instance, some dinoflagellates (e.g. *Noctiluca scintillansa*, *Prorocentrum shikokuense*, and *Karenia mikimotoi*) are found as eurythermal species [10], whereas some others (e.g. *Ceratium fusus*) are stenothermal species [11]. Generalists are typically more versatile but less efficient than specialists, which perform better in specific habitats [12]. However, under fluctuating conditions (e.g. climate warming and habitat disturbances) generalists often outcompete specialists. This tendency has been documented in a wide spectrum of organisms, including plants [13], coral reef fish [14], birds [15], mammals [16], and cyanobacteria [17]. A similar trend in dinoflagellates remains unexplored. Moreover, as one of the main factors that drive the dynamics of dinoflagellates, global eutrophication caused the loss of marine habitats (e.g. loss of seagrass meadows, seaweeds, coral reefs, and low-nutrient habitats), and thus lead to the decline in specialists [17]. We hypothesize that climate warming and eutrophication will promote the dominance of generalist dinoflagellates. Furthermore, due to their broader niche adaptability and concomitant influences of eutrophication, HDBs dominated by generalists may expand geographically under climate warming rather than simply follow the conventional view that HDBs migrate to higher latitudes.

Many studies on the effects of climate warming and eutrophication on dinoflagellate blooms rely on the data spanning several decades [18–20]. However, understanding long-term dynamics requires longer time series data. The Holocene consists of three stages (early Holocene: 11.7–8 kiloyear before present (kyr BP), middle Holocene: 8–4 kyr BP, and late Holocene: 4 kyr BP-present) and encompasses significant climate and environmental changes [21]. A previous study found the global surface temperature in low latitude regions (30°N to 30°S) exhibited a distinct increase from 11 to 5 kyr BP, and then levelled off [22]. Based on reconstructed mean annual sea-surface temperature (SST) by ${\mathrm{U}}_{37}^{K\prime }$ index in the northern South China Sea (SCS), a previous study found mean annual SST increased by ~3°C from ~11.5 to ~1.2 kyr BP [23]. During this period, relative sea level in the northern SCS rose from −50 m at ~10.5 kyr BP to the present height (~7.0 kyr BP), and has remained stable [24]. Additionally, human activities increased rapidly during the late Holocene in China [25, 26]. Therefore, analyzing dinoflagellate dynamics during the Holocene can help us understand how climate warming and nutrient enrichment (associated with increased human activities in late Holocene) have impacted dinoflagellate blooms.

Studying paleobiology for microorganisms like dinoflagellates is challenging, sedimentary ancient DNA (sedaDNA) extracted from marine sediment core provides an effective way to uncover palaeodinoflagellate community dynamics [27]. The low temperatures and anoxic conditions on the seafloor are conducive to preserving sedaDNA against degradation [28]. Recently, planktonic sedaDNA from marine sediments has been increasingly explored, providing valuable insights into ancient plankton communities [29, 30].

In this study, to address the above-mentioned hypothesis, we collected a marine sedimentary core from the northern SCS and surface sediments from various geographic locations. The dinoflagellate communities in core and surface sediment were analyzed using 18S rRNA gene amplicon sequencing, and further compared to contemporary global ocean dinoflagellate communities (18S rRNA gene data from *TARA Oceans*). The dinoflagellate data were then linked with recent 40 years of dinoflagellate bloom records (include date, location, and the main dinoflagellate species) in Chinese coastal waters. The aims of this study were to 1) reconstruct ancient dinoflagellate communities during the Holocene (~12 to ~0.8 kyr BP) in northern SCS; 2) uncover the effects of paleoclimate and paleoenvironmental changes on the succession of ancient generalist and specialist dinoflagellates; 3) combine with contemporary global dinoflagellate community data and Chinese records of 40-year′s dinoflagellate blooms to further understand the impacts of global climate warming and eutrophication on dinoflagellate blooms.

## Materials and methods

### Sediment sampling and ^14^C dating

**Figure 1:**
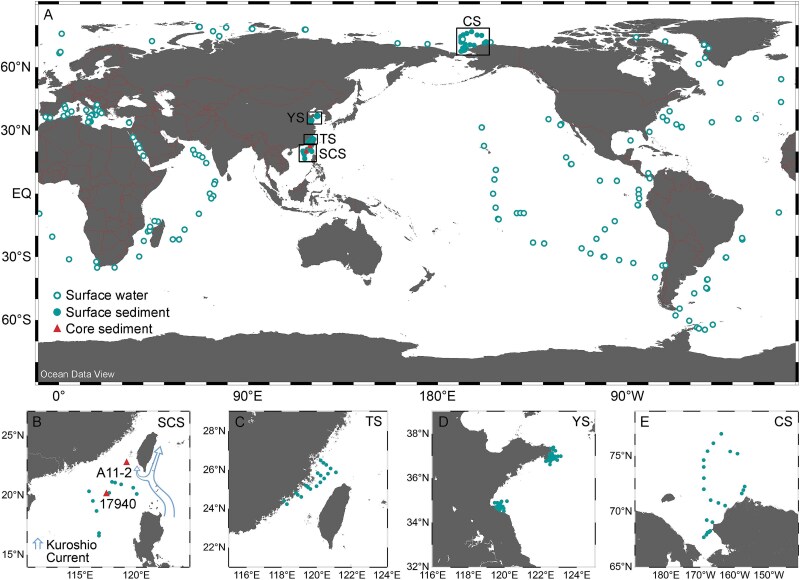
Study sites (A) and sampling stations in the South China Sea (B, SCS. The blue arrows indicate the main axis and a branch of Kuroshio current), Taiwan Strait (C, TS), Yellow Sea (D, YS), and Chukchi Sea (E, CS).

The contemporary surface sediment samples were collected in the Chukchi Sea (CS, July 2014, 25 stations), the Yellow Sea (YS, July 2021, 57 stations), the Taiwan Strait (TS, July 2021, 19 stations), and the South China Sea (SCS, July 2021, 11 stations). Two duplicate samples were sequenced for one station in CS, TS, and SCS, but one sample was sequenced for one station in YS (Fig. 1). Surface sediment samples (0–2 cm) were collected using a grab sampler and frozen immediately after sampling.

The sediment gravity core A11–2 (ancient sediment) was collected using the 13.540 gravity corer Ø101,6 mm (KC Denmark A/S, Denmark) during the NSFC (National Natural Science Foundation of China) shared cruise of Tawan Strait in July 2021 in northern SCS at intermediate water depths (119° 17′ 9.996″ E, 22° 7′ 0.012″ N, 1300 m depth) (Fig. 1A and B). The cores contained undisturbed hemipelagic sediments with high sedimentation rates.

The total length of the core A11–2 is 320 cm, and it covers the last ~17 kyr including the whole Holocene (the last ~11.7 kyr) according to ^14^C dating (Fig. S1). Core sediment samples were sliced into 1-cm section samples and frozen immediately. Five sediment section samples were evenly selected by 50-cm interval from core A11–2 (1, 50, 100, 150, and 200 cm), and well-preserved dominant foraminifera (*Globorotalia Menardii* and *Orbulina universa*) of ~10 mg were picked from each sediment section sample under a stereomicroscope. The picked foraminifera samples were dated for ^14^C ages using accelerator mass spectrometry system [23]. The standard error of the ^14^C dating was estimated at ±30 yr in each selected layer.

### Analyses of biomarkers in core A11–2

Dinosterol was used as a biomarker for dinoflagellate and the odd C number long-chain (C27 + C29 + C31) *n-*alkanes was used as a biomarker for terrestrial input (TI) as previously described [31]. Odd C number long-chain *n-*alkanes are the main component of higher plant wax and have been widely used to trace TI [32, 33]. Dinosterol is a well-established biomarker for dinoflagellates, and its concentration in core sediment correlates well with the production rate of dinoflagellates [34]. Additionally, sediment alkenones were also analyzed for mean annual SST reconstruction in northern SCS during the Holocene.

Based on ^14^C dating data, 171 samples were selected for biomarker analyses (1–149 cm (0.8–4.6 kyr BP): samples were selected by 2-cm interval; 150–249 cm (4.6–12 kyr BP): samples were selected by 1-cm interval). The *n-*alkanes, *n-*alkenones, and sterols were extracted, purified, and isolated following protocols from our previous study [35]. The purified *n-*alkanes, *n-*alkenones, and sterols were identified using a thermo gas chromatography–mass spectrometry (GC–MS) system. Quantification of these biomarkers was performed using an Agilent 7890B GC.

The biomarker alkenone unsaturation index (${\mathrm{U}}_{37}^{K\prime }$) is a widely used proxy for determining past SST. The ${\mathrm{U}}_{37}^{K\prime }$ index is based on a highly resistant group of organic molecules with long carbon chains, and a characteristic pattern and different numbers of double bonds known as alkenones. Biosynthesis of these compounds is restricted to several ubiquitous species of haptophyte algae and is dependent on water temperature [36]. We calculated the ${\mathrm{U}}_{37}^{K\prime }$ values following the equation defined by Prahl and Wakeham (${\mathrm{U}}_{37}^{K\prime }=\left[{C}_{37:2}\right]/\left[{C}_{37:2}+{C}_{37:3}\right]$) [37]. Then, the mean annual SST was reconstructed using the equation: $SST=\left({\mathrm{U}}_{37}^{K\prime }-0.044\right)/0.033,$ which has been verified by a global calibration based on 370 core-top samples from open ocean sites [38].

### Sequencing of micro-eukaryotic 18S rRNA genes in surface and core (A11–2) sediment samples

Total DNA in 1 g wet sediment was extracted using a FastDNA SPIN Kit (MP Biomedicals, USA) following the manufacturer′s instructions. In total, 171 samples were selected in core A11–2 (same as the biomarker samples) and 167 samples were selected in surface sediment. The V4 region of eukaryotic 18S rRNA genes were amplified by PCR using the primer pairs 528F (5′-GCG GTA ATT CCA GCT CCA A-3′) and 706R (5′-ATT CCR AGA ATT TCA CCT CT-3′) [39, 40], according to our previous study (triplicated PCRs for each sample were performed) [41]. The triplicate PCR products were pooled and gel purified. Sequencing libraries were generated from the pooled PCR products and sequenced on a HiSeq System (Illumina, USA).

### Micro-eukaryotic 18S rRNA gene sequence data in contemporary water samples in global oceans

The micro-eukaryotic 18S rRNA gene V4 region sequence data from contemporary water samples were obtained from the *Tara Oceans* project [42]. Briefly, water samples were collected from 398 stations in the open ocean, covering the Arctic, Atlantic, Indian, East Pacific, and Southern Oceans, as well as the Mediterranean and Red Seas (Fig. 1A). We selected only surface water samples (~5 m deep). At some stations, different fractions of plankton body size were collected: 0.8–5 μm, 5–20 μm, 20–180 μm, and 180–2000 μm. We did not classify the water samples from contemporary global oceans by plankton body size because we did not distinguish the body size of micro-eukaryotes in our sediment samples. In total, 677 water samples were used in this study (Fig. 1A). The 18S-V4 rRNA genes were PCR-amplified and sequenced on a HiSeq System (Illumina). All sequence data were retrieved from the European Nucleotide Archive under accession numbers PRJEB6610 and PRJEB9737.

### Bioinformatics

The open-source R package DADA2 was used to filter the low-quality sequences with default settings following the DADA2 Pipeline Tutorial 1.16 (http://benjjneb.github.io/dada2/tutorial.html) [43]. Specifically, the nucleotides at the end of the reads with mean quality score < 30 were trimmed. Then, reads were further filtered (maxN = 0, maxEE = c(2,2), truncQ = 2) and the error rates were further reduced by DADA2 core sample inference algorithm. Filtered reads were merged (~310 bp) and the chimeric sequences were removed using removeBimeraDenovo function in R package. DADA2 uses amplicon sequence variants (ASVs) instead of operational taxonomic units (OTUs) to obtain the taxonomic diversity of microorganisms. The remaining high-quality sequences were annotated by the Protist Ribosomal Reference (PR2) version 4.14.0. Bacterial, mammal, fish, plant, and unknown sequences were removed manually from the sequence data based on the PR2 taxonomic analysis result. Then, we used a subset of 26 245 sequence reads (the minimum read number in all core samples) randomly selected from each sample to normalize sequencing efforts. The rarefaction curves tended to saturate after 25 000 reads indicating the micro-eukaryotic communities were well-sampled (Fig. S2). The raw sequence data from sediment samples have been deposited in the Genome Sequence Archive (National Genomics Data Center, China National Center for Bioinformation / Beijing Institute of Genomics, Chinese Academy of Sciences) (https://ngdc.cncb.ac.cn/gsa/) with the accession numbers CRA007427, CRA018265, and CRA018173.

### Quantitative PCR (qPCR) of 18S rRNA genes in core A11–2

To quantify the copy number of micro-eukaryotic 18S rRNA genes in core A11–2, qPCR was performed using SYBR Green I for the DNA extracts. Reactions were performed in a final volume of 12 μl using 6 μl SYBR Premix Ex Tap, 2 μl of DNA, and 0.5 μl 18S rRNA gene primer pairs (the same primer pairs as used in 18S rRNA gene amplicon sequencing). Thermal cycling was performed with 40 cycles of denaturation at 95°C for 30 s, annealing at 55°C for 30 s, and extension at 72°C for 90 s.

### Detection of generalist, specialist and non-significant dinoflagellates in core A11–2

To identify generalist, specialist and non-significant dinoflagellate ASVs in core A11–2, the "EcolUtils" package in R were used, based on the deviation of niche width index occurrence (https://github.com/GuillemSalazar/EcolUtils). Briefly, ASVs were randomly shuffled 1000 times via occurrence index and the simulated niche breadth was calculated. ASVs with observed niche breadth greater than 95% of the simulated niche breadth were classified as generalist dinoflagellates, whereas those with observed niche breadth lower than 5% were classified as specialist dinoflagellates. In addition, ASVs belonged to neither generalists nor specialists (their observed niche breadth ≥5% of the simulated niche breadth and ≤ 95% of the simulated niche breadth) were classified as non-significant dinoflagellates.

### Construction of co-occurrence networks between dinoflagellate and microbial eukaryotic ASVs in core A11–2

Co-occurrence networks between dinoflagellate and microbial eukaryotic ASVs in core A11–2 were constructed based on Spearman′s rank correlations using the "picante" R package [44]. To reduce noise and thus false-positive predictions, the ASVs occurring in fewer than five samples were excluded. Only robust (*r* > |0.4|) and statistically significant (*P* < 0.01) correlations were included in the networks [45]. Network visualizations were generated using Gephi version 0.9.1.

### Data sources of sea-surface salinity in northern SCS during Holocene, Kuroshio current intensity index, Chinese archaeological sites, and historical population

Data of sea-surface salinity (SSS) were obtained from a previous study on core 17940 of northern SCS [23] (Fig. 1B). Briefly, the SSS was estimated using a transfer equation [46], subtracting past variations in SST and global ice-volume from the planktonic δ^18^O signal of *G. ruber* (white). The SSS data are available through the open access PANGAEA Data Publisher for Earth & Environmental Science (https://www.pangaea.de/).

Kuroshio intensity index was sourced from a previous study [47], which collected two gravity cores: one beneath the main axis of the Kuroshio Current (northeastern Taiwan) and the other east of Tsushima Current, a major branch of the Kuroshio Current. The study identified and counted planktonic foraminifera with sizes ical pμm, using *Pulleniatina obliquiloculata* (%) as an indicator species for Kuroshio Current strength. *P*. *obliquiloculata* is a powerful indicator of the Kuroshio Current variability in many studies, because in the sediments of East China Sea, *P*. *obliquiloculata* is very abundant (exceeding 10% of the planktonic foraminiferal fauna) beneath the main axis of the Kuroshio Current in East China Sea sediments [48, 49]. The two cores showed a similar tendency for the Kuroshio Current strength, and we chose the core in east of Tsushima Current due to its higher resolution.

Data on archaeological sites in China were obtained from a previous study [26]. These data were extracted from 39 volumes of Atlas of Chinese Cultural Relics, covering most of mainland Chinese, and are available through PANGAEA (https://www.pangaea.de/). Historical population data for China were taken from a previous study [25], which summarized population data from two studies [50, 51], both providing comprehensive analyses of Chinese population.

### Recent records of harmful dinoflagellates in Chinese coastal regions

Five recent references [52–56] summarized the harmful dinoflagellate species that occurred in Chinese coastal regions in recent 40 to 60 years, which revealed 115 known major harmful dinoflagellate species. These dinoflagellate species belong to 36 genera (i.e. defined as potentially harmful dinoflagellate genera in this study). We further screened the dinoflagellate ASVs in core A11–2 for taxa that belong to the 36 genera using the PR2 taxonomic database, and analyzed the dynamics of potentially harmful dinoflagellate genera (Table S1).

**Figure 2:**
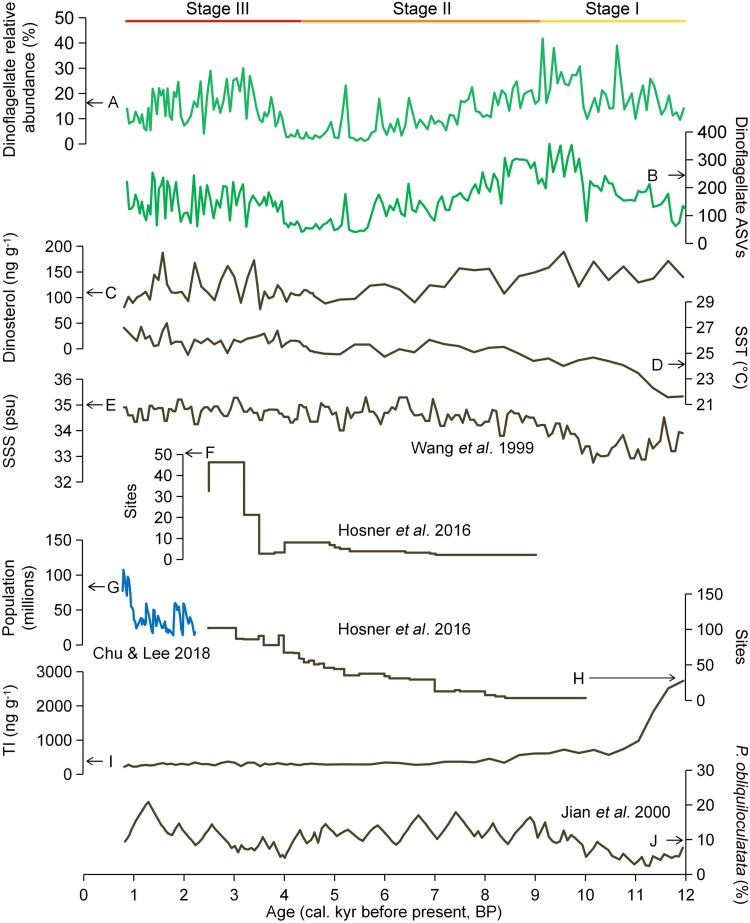
Variations in dinoflagellate relative abundance (RAB) and species richness (SRI) along with paleoenvironmental factors. A) RAB of dinoflagellates (% dinoflagellate sequences over total micro-eukaryotic sequences); B) SRI of dinoflagellates estimated by dinoflagellate ASV number; C) dinoflagellate abundance indicated by dinosterol concentration (A11–2); D) mean annual sea-surface temperature (SST, A11–2); E) sea-surface salinity (SSS, core 17940); F) variations in the number of archaeological sites per 1000 km^2^ in southern China; G) historical Chinese population; H) variations in the number of archaeological sites per 1000 km^2^ in northern China; I) terrestrial input (TI, A11–2) during the Holocene in the northern South China Sea (SCS); J) variations in Kuroshio intensity index (abundance of *Pulleniatina obliquiloculata* to abundance of total foraminifer) during the Holocene in the northwestern Pacific Ocean.

### Recent records of harmful dinoflagellate blooms in Chinese coastal regions

The data on harmful dinoflagellate bloom records (1980–2023) in Chinese coastal regions were sourced from a previous study [57]. This study compiled harmful algal bloom records from two books covering 1980–2009, annual reports from the State Oceanic Administration of China and its branches, Ministry of Environmental Protection of China, and local governments from 11 coastal administrative areas in China (2010–2015). We further screened dinoflagellate bloom records from this study, and added bloom records from 2016 to 2023 using data from the Bulletin of Marine Disasters of China (2016–2023, State Oceanic Administration of China. https://www.mnr.gov.cn/sj/sjfw/hy/gbgg/zghyzhgb/). These records include bloom locations and dominant species.

After 1980, systematic monitoring for harmful algal blooms (HABs) was established by the State Oceanic Administration of China, which includes routine monitoring, contingent monitoring, and tracking monitoring. Monitoring was conducted by ship, with assistance from aircrafts, satellites, and fishermen′s reports. According to China′s Technical Specification for Red Tide Monitoring (HY/T 069–2005), routine monitoring includes four surveys per month during seasons with high frequency, and one to two surveys per month during other seasons. Contingent monitoring involves surveys at 3-day intervals when HABs are expected to occur. Tracking monitoring is conducted daily when blooms are detected or ongoing. Monitoring reports include the location, date, and dominant species of HABs [57].

## Results

### Temporal variation of dinoflagellates, palaeoenvironmental factors and human activities during the Holocene

Based on the dinoflagellate community profile, core A11–2 was divided into three stages: ~12 to ~9.1 kyr BP (stage I), ~9.1 to ~4.3 kyr BP (stage II), and ~ 4.3 to ~0.8 kyr BP (stage III) (Fig. 2). The relative abundance (% dinoflagellate sequences over total micro-eukaryotic sequences; relative abundance [RAB]) and species richness (estimated by ASV numbers; species richness [SRI]) of dinoflagellates increased in stage I, then decreased in stage II, and increased significantly again in stage III (Fig. 2A and B). Moreover, dinosterol concentration showed significant correlations with the RAB of dinoflagellates in the core (Figs. 2C and S3). The variation in the copy number of the dinoflagellate 18S rRNA genes (dinoflagellate RAB × copy number of total 18S rRNA genes) was consistent with the RAB of dinoflagellates in A11–2 (Fig. S4).

At the A11–2 site, the mean annual SST exhibited an increasing trend during the period represented by the core A11–2 in northern SCS (Fig. 2D). It rose rapidly from 21.63°C to 24.49°C in stage I with an increase of 2.86°C, then increased steadily to 25.91°C, at an increment of 1.42°C, in stage II, and finally climbed slowly to 26.97°C with an increase of 1.06°C in stage III.

According to core 17940 from northern SCS there was a short period of SSS decline, from 33.91 psu in ~12 kyr BP to 32.76 psu in ~10.2 kyr BP, followed by a quick rise to 35.30 psu in the period from ~10.2 kyr BP to ~5.6 kyr BP. Finally, the SSS stabilized around 34.71 psu (~5.6 to ~0.8 kyr BP) (Fig. 2E).

Evidence of increasing human activities was found from the increase of archaeological sites in China, the important relics of human history, which changed with Chinese human populations. In southern China, a two-step increase of archaeological sites was observed: sites per 1000 km^2^ increased from ~3 (~5.3 kyr BP) to ~10 (~4.9 kyr BP), and further to ~47 (~3.2 kyr BP) (Fig. 2F). In northern China, the archaeological sites per 1000 km^2^ increased gradually from ~3 (~10 kyr BP) to ~35 (~5.2 kyr BP), an increment of 32 sites, followed by a rapid increase from ~35 (~5.2 kyr BP) to ~102 (~2.2 kyr BP), an increment of 67 sites (Fig. 2H). Additionally, the historical population in China increased from ~18 to ~77 million (~2.2 to ~0.8 kyr BP) (Fig. 2G).

Terrestrial input (TI) changed throughout the period covered in this study. The TI decreased quickly in stage I, from 2732 to 611 ng g^−1^, then 270 ng g^−1^ in stage II, and stabilized at around 290 ng g^−1^ in stage III (Fig. 2I). The Kuroshio Current intensity (indicated by *P*. *obliquiloculata* %) increased from ~12 to ~7.4 kyr BP, then decreased from ~7.4 to ~4.0 kyr BP, but rose again after ~4.0 kyr BP (Figs. 2J and 1B).

### Correlation between dinoflagellates and palaeoenvironmental factors in core sediment

**Figure 3:**
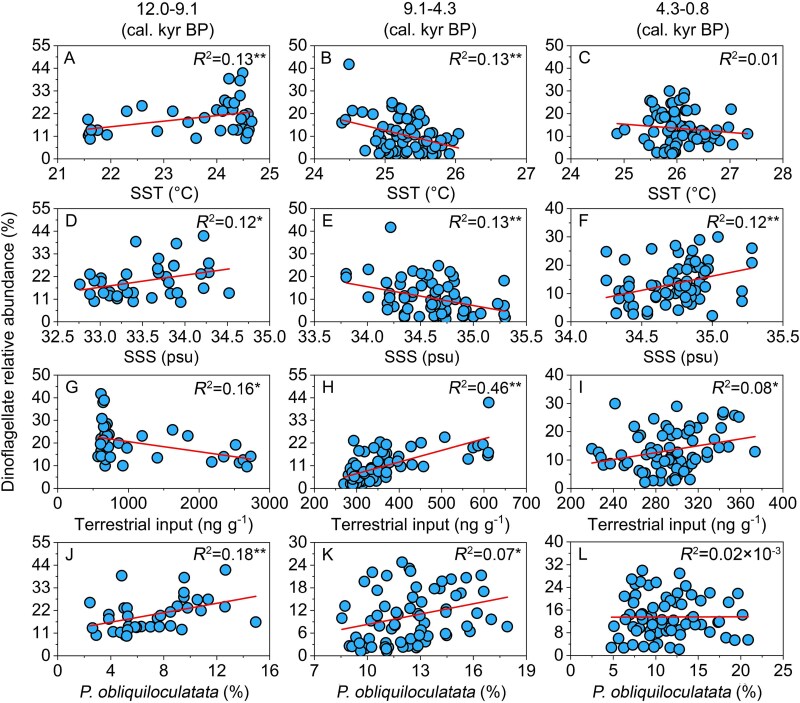
Linear regression showing the relationships between dinoflagellate RAB in core A11–2 and palaeoenvironmental factors during the periods from ~12 to ~9.1 kyr BP (A, D, G, J), ~9.1 to ~4.3 kyr BP (B, E, H, K), and ~ 4.3 to 0.8 kyr BP (C, F, I, L). ^*^ - *P* < 0.05, ^**^ - *P* < 0.01. *P. obliquiloculatata,* the formaminifera *Pulleniatina obliquiloculata.* More *P*. *obliquiloculatata* indicates higher Kuroshio current intensity.

In stage I of core A11–2, the RAB and SRI of dinoflagellates exhibited significant correlations (*P* < 0.05) with SST (positive), SSS (positive), TI (negative), and Kuroshio Current intensity (positive). In stage II, these correlations (*P* < 0.05) changed directions (negative with SST, negative with SSS, and positive with TI) except for the correlation between RAB as well as SRI and Kuroshio Current intensity. In stage III, only SSS and TI showed significant positive (but weakly) correlations with RAB of dinoflagellates (Figs. 3 and S5).

### Temporal variation of generalist and specialist dinoflagellate ASVs in core sediment as well as the distribution of these ASVs in contemporary surface sediment and water

**Figure 4:**
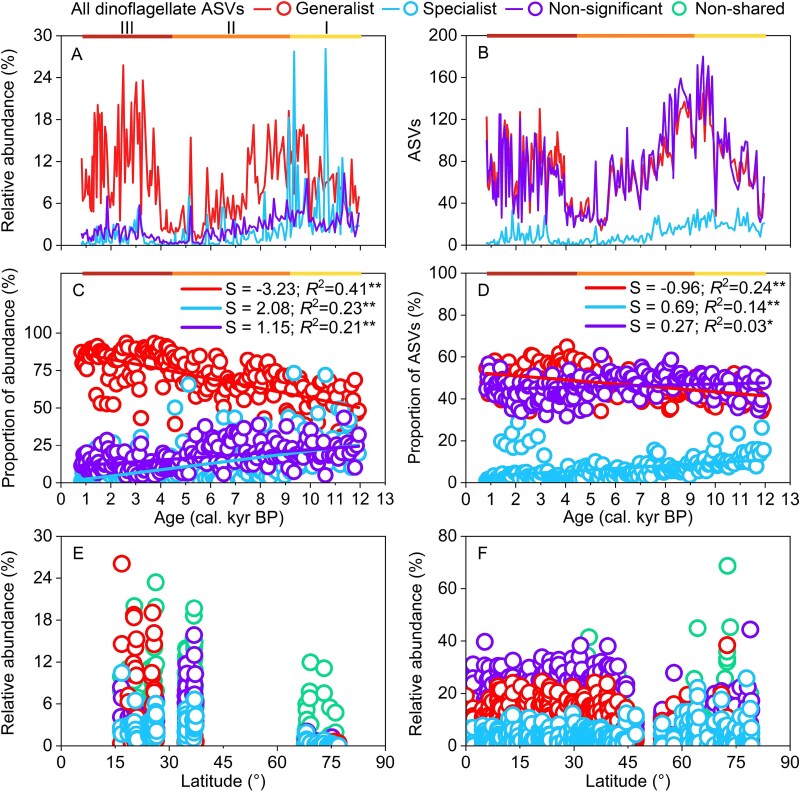
Abundance and diversity of generalist, specialist, and non-significant dinoflagellates. A) RAB. B) SRI. C) proportion of abundance estimated by dinoflagellate RAB in a sub-group divided by total dinoflagellate RAB. D) proportion of SRI estimated by dinoflagellate SRI in a sub-group divided by total dinoflagellate SRI. E-F) variation in RAB of shared generalist, specialist, and non-significant dinoflagellate ASVs in contemporary surface sediment (E) and surface water (F).

To gain insights into how generalists, specialists, and the rest (non-significant) among dinoflagellates responded to climate and environmental conditions differently, we analyzed their trends of change. In stage I, the RAB of generalist, specialist, and non-significant dinoflagellate ASVs increased. In stage II, the RAB of all three types of dinoflagellate ASVs decreased; however, the decline of generalist dinoflagellates lagged behind that of specialist and non-significant dinoflagellates, with an obvious decline in generalists occurring after 7.7 kyr BP. In stage III, the RAB of generalist dinoflagellate ASVs increased significantly after 4.0 kyr BP, although the increase in specialist and non-significant dinoflagellate ASVs was moderate (Fig. 4A). Similarly, in stage I, the number of generalist, specialist, and non-significant dinoflagellate ASVs increased. In stage II, the number of all three types decreased. In stage III, the number of all three types increased again, but the increase in specialist dinoflagellates was less pronounced (Fig. 4B). Moreover, the proportion of generalist dinoflagellate abundance (generalist dinoflagellate RAB divided by total dinoflagellate RAB) significantly increased from stage I to stage III, whereas the proportion of specialist and non-significant dinoflagellate abundance both significantly decreased (Fig. 4C). Similarly, the proportion of generalist SRI (generalist dinoflagellate SRI divided by total dinoflagellate SRI) significantly increased from stage I to stage III, although the proportion of specialist and non-significant dinoflagellate SRI significantly decreased (Fig. 4D).

Many generalist, specialist, and non-significant dinoflagellate ASVs found in cores were also found in contemporary surface sediment and surface water. These shared generalists were more abundant than the shared specialists in low and middle latitude surface sediment and surface water, whereas shared specialists had comparable RAB to generalists in higher latitude regions (Fig. 4E and F). Specifically, for contemporary surface water, the mean RABs of shared generalist dinoflagellate ASVs versus shared specialist dinoflagellate ASVs was 5.9% vs 2.3% in less than latitude of 42°. However, the mean RABs of shared generalist dinoflagellate ASVs versus shared specialist dinoflagellate ASVs was 2.7% vs 4.6% in greater than latitude of 42° (Fig. 4F).

### Occurrence frequency and family composition of generalist, specialist, and non-significant dinoflagellate ASVs in core sediments and their correlations with palaeoenvironmental factors

In general, the mean occurrence frequency (mean number of sites occupied by ASVs) of generalist dinoflagellate ASVs (20.44) was markedly higher than that of non-significant (1.33) and specialist ASVs (1.29) in core A11–2 (Fig. S6A). The occurrence frequency of generalist (*R*^2^ = 0.30, *P* < 0.01), specialist (*R*^2^ = 0.72, *P* < 0.01) and non-significant ASVs (*R*^2^ = 0.58, *P* < 0.01) was significantly positively correlated with their mean RABs (Fig. S6B). During the Holocene of northern SCS, the generalist ASVs (3.66) had significantly lower mean variation coefficient than the non-significant (11.43) and specialist ASVs (12.00) (Fig. S6C).

The RAB of specialist dinoflagellate ASVs showed a significant correlation with SST, SSS, TI, and Kuroshio Current intensity (*P* < 0.05). This was true also for the RAB of non-significant dinoflagellate ASVs, except for Kuroshio Current intensity. However, RAB of generalist dinoflagellate ASVs did not correlate with any palaeoenvironmental factors (*P* > 0.05) (Fig. S6D).

Generalist dinoflagellates had the highest RAB in Syndiniales-group I (6.9%), Syndiniales-group II (5.6%), Gymnodiniales (1.0%), and Prorocentrales (0.5%). In contrast, specialist dinoflagellates had the highest RAB in Syndiniales-group I (1.7%), Peridiniales (0.3%), Syndiniales-group II (0.3%), and Dinophysiales (0.3%). For non-significant dinoflagellates the highest RAB occurred in Syndiniales-group I (1.2%), Syndiniales-group II (0.9%), Suessiales (0.3%), and Gymnodiniales (0.2%) (Fig. S6E).

### Temporal variation of dinoflagellate ASVs that belong to the potentially harmful dinoflagellate genera in core sediments and the distribution of these ASVs in contemporary surface sediments and waters

We analyzed the dynamics of generalist, specialist, and non-significant dinoflagellate ASVs that belong to the potentially harmful dinoflagellate genera in core A11–2. We examined the 36 potentially harmful dinoflagellate genera that occurred in Chinese coastal regions in recent 40–60 years, and found that 16 of them were abundantly represented in core A11–2.

**Figure 5:**
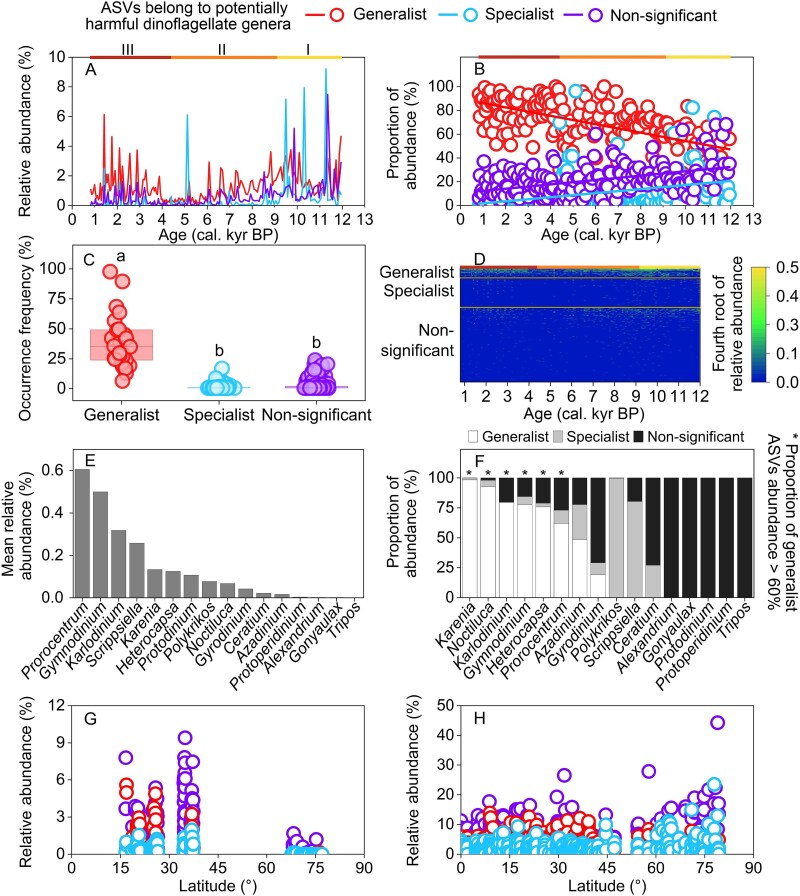
RAB (A), proportion of abundance (dinoflagellate RAB in a sub-group divided by RAB of total potentially harmful dinoflagellate genera) (B) and occurrence frequency (C) of generalist, specialist, and non-significant dinoflagellate ASVs belonging to the 16 potentially harmful dinoflagellate genera in core A11–2. Individual RAB of generalist, specialist, and non-significant dinoflagellate ASVs belonging to the 16 genera in core A11–2 (D). Mean RAB of the 16 genera in core A11–2 (E). Proportion of abundance of generalist, specialist, and non-significant ASVs in each genus in core A11–2 (F). Variations in RAB of shared generalist, specialist, and non-significant dinoflagellate ASVs belonging to the 16 genera in contemporary surface sediment (G) and surface water (H).

The RABs of generalist, specialist, and non-significant dinoflagellate ASVs from these 16 genera were high in stage I, but relatively low in stage II. In stage III, the RAB of generalists belonging to the 16 genera increased significantly after 4.0 kyr BP, whereas the RABs of specialists and non-significant dinoflagellates showed smaller increases (Fig. 5A). In addition, the proportion of abundance for generalist dinoflagellate ASVs belonging to the 16 genera significantly increased from stage I to stage III (*R*^2^ = 0.34, *P* < 0.01) (generalist dinoflagellate RAB divided by RAB of total potentially harmful dinoflagellate genera), whereas the proportion of abundance for specialist (*R*^2^ = 0.12, *P* < 0.01) and non-significant (*R*^2^ = 0.19, *P* < 0.01) dinoflagellates significantly decreased (Fig. 5B). Moreover, in core A11–2, the occurrence frequency of generalist ASVs from these 16 genera was the highest (38.60 sites), followed by non-significant (1.38 sites) and specialist ASVs (2.49 sites) (Fig. 5C). Generalist ASVs from these 16 genera occurred frequently in almost all layers of core A11–2. In contrast, although some specialist and non-significant ASVs from these genera had high RABs in certain layers (especially at the bottom), they were replaced by other ASVs in other layers (Fig. 5D).

Among the 16 potentially harmful dinoflagellate genera detected in core A11–2, the genus *Prorocentrum* had the highest mean RAB (0.6%), followed by *Gymnodinium* (0.5%), *Karlodinium* (0.3%), *Scrippsiella* (0.3%), and *Karenia* (0.1%) (Fig. 5E). Among the ASVs belonging to genus *Karenia*, generalist ASVs accounted for 98% of abundance, followed by *Noctiluca* (93%), *Karlodinium* (80%), *Gymnodinium* (78%), *Heterocapsa* (75%), and *Prorocentrum* (63%) (Fig. 5F).

Many generalist, specialist, and non-significant ASVs from these 16 genera found in core sediment were also present in contemporary surface sediment and surface water. These shared generalists had higher RAB than the shared specialists in low and middle latitude surface sediments and surface waters (with higher SST); however, in higher latitude regions (with lower SST), shared specialists had comparable RABs to the generalists (Figs. 5G, H, and  S7).

### Comparison of potentially harmful dinoflagellate genera in core sediments with dominant dinoflagellate blooms in Chinese coastal waters from 1980 to 2023

**Figure 6:**
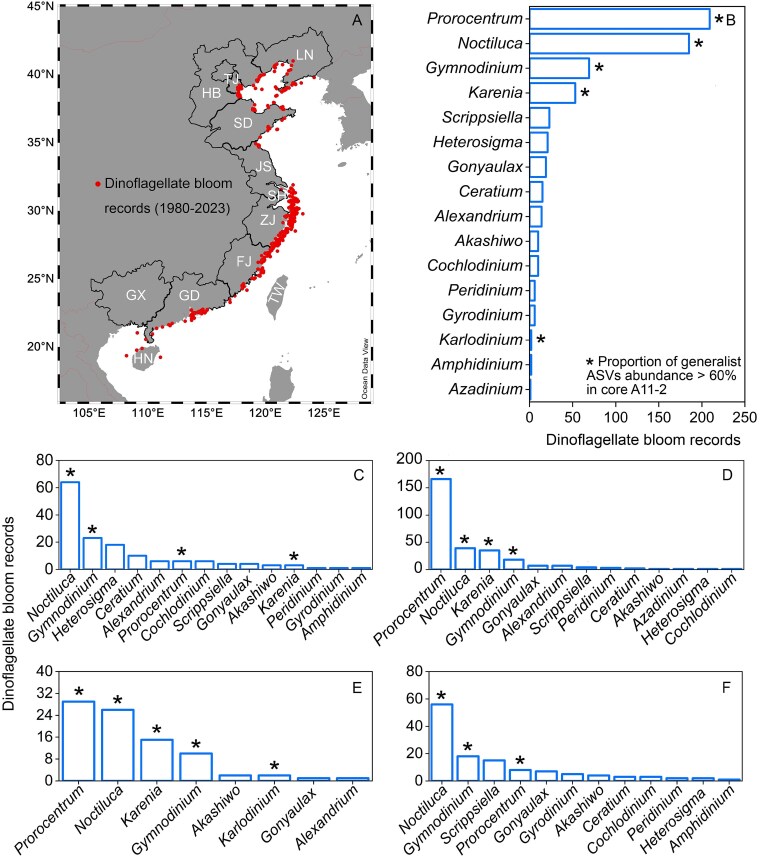
Dinoflagellate bloom records from 1980 to 2023 in Chinese coastal regions (A). Main dinoflagellate genera in the dinoflagellate bloom records in all Chinese coastal regions (B), in LN, TJ, HB, SD, and JS (C), in SH and ZJ (D), in FJ (E), in GD, GX, and HN (F).

According to the records of dinoflagellate blooms in Chinese coastal regions from 1980 to 2023 (Table S2), the highest number of dinoflagellate bloom records was found in the coastal regions from Shanghai (SH) to Zhejiang (ZJ) (SH + ZJ, 266 records), followed by the coastal regions from Liaoning (LN) to Jiangsu (JS) (LN + TJ + HB + SD + JS, 134 records), regions from Guangdong (GD) to Hainan (HN) (GD + GX + HN, 114 records) and Fujian (FJ) (76 records) (Fig. 6A).

The dominant dinoflagellate genera reported in bloom records along Chinese coastal regions were *Prorocentrum* (209 records), *Noctiluca* (185 records), *Gymnodinium* (69 records), and *Karenia* (53 records) (Fig. 6B). Moreover, the main dinoflagellate genera in bloom records for region LN + TJ + HB + SD + JS included *Noctiluca*, *Gymnodinium*, *Heterocapsa*, and *Ceratium* (Fig. 6C). In region SH + ZJ, the dominant dinoflagellate genera were *Prorocentrum*, *Noctiluca*, *Karenia*, and *Gymnodinium* (Fig. 6D). Similarly, in region FJ, the principal dinoflagellate genera were *Prorocentrum*, *Noctiluca*, *Karenia*, and *Gymnodinium* (Fig. 6E). The main genera reported in bloom records for region GD + GX + HN were *Noctiluca*, *Gymnodinium*, *Scrippsiella*, and *Prorocentrum* (Fig. 6F). Moreover, the most typical generalist genera (>60% generalist ASVs abundance) in core A11–2 (Fig. 5F) were also taxa contributing to the most frequent bloom records from south to north of China (Fig. 6C–F).

### Homogenization of beta-diversity for dinoflagellate communities

**Figure 7:**
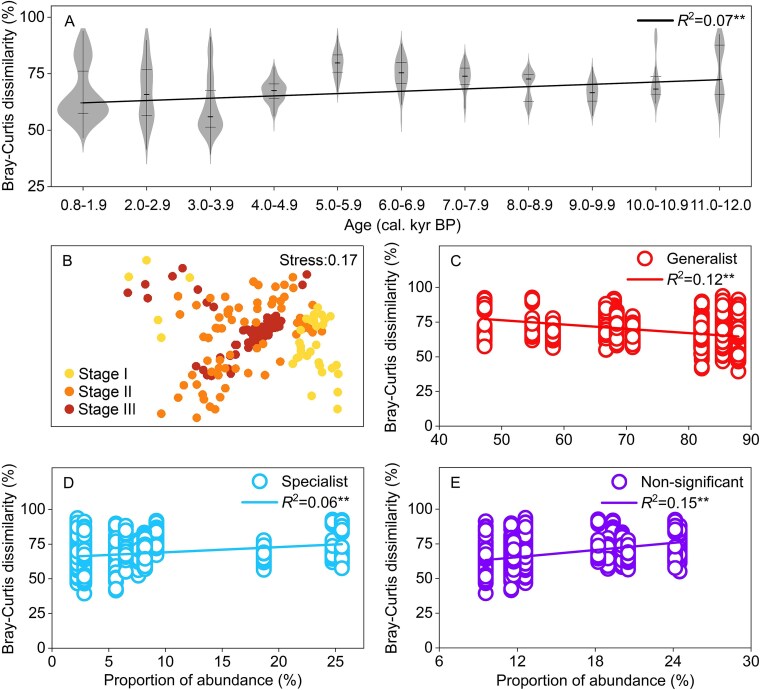
Variation in dissimilarity of dinoflagellate communities within windows of ~1000 years in core A11–2 (A). MDS ordination showing the variations in dinoflagellate community composition in core A11–2 (B). Correlations between dissimilarity of dinoflagellate communities and median in proportion of abundance of generalist (generalist dinoflagellate RAB divided by total dinoflagellate RAB) (C), specialist (D), and non-significant (E) dinoflagellate ASVs per ~1000 year in core A11–2.

We calculated the mean dissimilarities among samples within windows of ~1000 years and we found the mean dissimilarities were gradually decreased from stage I to stage III (*R*^2^ = 0.07, *P* < 0.01) indicating the increased homogenization of beta-diversity of dinoflagellate communities from early to late Holocene of northern SCS (Fig. 7A). MDS ordination also showed that the dissimilarities in dinoflagellate community composition within samples were lower in stage I and II than in stage III (Fig. 7B). Moreover, with the proportion of generalist dinoflagellate abundance (generalist dinoflagellate RAB divided by total dinoflagellate RAB) increased, mean dissimilarities among samples significantly decreased (Fig. 7C). In contrast, as the proportion of specialist and non-significant dinoflagellate abundance increased, mean dissimilarities among samples significantly increased (Fig. 7D and E).

### Co-occurrence networks between dinoflagellate and microbial eukaryotic ASVs

**Figure 8:**
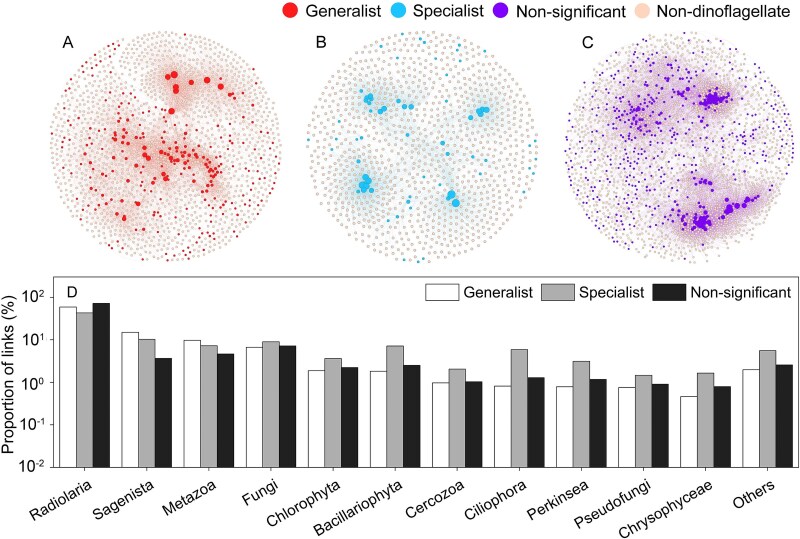
Co-occurrence networks between dinoflagellate and other microbial eukaryotic ASVs for generalist (A), specialist (B), and non-significant (C) dinoflagellates. Composition of phylum level of other microbial eukaryotes in the co-occurrence networks (D).

Co-occurrence networks between generalist dinoflagellate ASVs and microbial eukaryotic ASVs exhibited an average degree of 8.42 (Fig. 8A). The average degree was 6.28 for networks between specialist dinoflagellate ASVs and microbial eukaryotic ASVs (Fig. 8B), and 13.1 for networks between non-significant dinoflagellate ASVs and microbial eukaryotic ASVs (Fig. 8C). The links in generalist network were dominated by four types: links between generalist dinoflagellates and Radiolaria (59.3%), Sagenista (14.9%), Metazoa (9.7%), and Fungi (6.7%). Similarly, links in the non-significant network were also dominated by these four types: Radiolaria (72.1%), Sagenista (3.7%), Metazoa (4.6%), and Fungi (7.2%). However, links in specialist network were diverse: Radiolaria (43.2%), Sagenista (10.2%), Fungi (9.0%), Metazoa (7.2%), Bacillariophyta (7.1%), Ciliophora (5.9%), Chlorophyta (3.6%), and Perkinsea (3.1%) (Fig. 8D).

## Discussion

### Dominant effects of paleoclimate on Holocene dinoflagellate relative abundance and diversity

Based on the relative abundance (RAB) and species richness (estimated by ASV number, SRI) of dinoflagellates in core A11–2, three stages were detected in northern SCS since Holocene. Stage I (~12 to ~9.1 kyr BP): RAB and SRI of dinoflagellates quickly increased. Stage II (~9.1 to ~4.3 kyr BP): RAB and SRI of dinoflagellates decreased quickly. Stage III (~4.3 to ~0.8 kyr BP): RAB and SRI of dinoflagellates increased significantly again (Fig. 2). The three stages are generally equivalent to the early (11.7–8 kyr BP), middle (8–4 kyr BP), and late (4 kyr BP - present) Holocene. This long-term dynamics of dinoflagellate DNA-based data were substantiated by the dinoflagellate lipid biomarker (dinosterol) in A11–2. DNA theoretically has a weaker long-term preservation potential than lipid biomarkers because it requires stricter preservation conditions, such as rapid burial of sediment, low temperature and oxygen [58]. However, lipid biomarkers are limited in taxonomic resolution. When used in combination, these methods complement each other. In this study, the congruence between their analysis results indicates our sedaDNA is reliable and compatible with conventional paleogeography.

In the early Holocene, following the end of the Younger Dryas cold event (~12.5–11.5 kyr BP), global temperatures increased quickly [21]. The rapid rise of mean annual SST in the stage I of Holocene in northern SCS likely promoted dinoflagellate growth, leading to an increase in RAB of dinoflagellates. Moreover, the intensity index for the Kuroshio Current increased in the stage I. With rising temperatures, relative sea levels in northern SCS rose quickly from −50 m (~10.5 kyr BP) to present-day levels (~7.0 kyr BP) [24]. This rise opened the Taiwan Strait, allowing seawater from the SCS to flow into the East China Sea and forming a branch of Kuroshio Current that flowed westward along the northern shelf margin of the SCS (crossing the regions of core A11–2, Fig. 1B). This Kuroshio intrusion brought warm water and nutrients that could promote phytoplankton growth near core A11–2. For example, a previous study found phosphate transported by Kuroshio intrusion promoted harmful dinoflagellate blooms (mainly *Karenia mikimotoi* and *P*. *shikokuense*) in the East China Sea near the Changjiang River estuary [59].

In stage II, as mean annual SST continued to rise in the northern SCS, RAB and SRI of dinoflagellates declined rapidly. We found evidence that some dinoflagellates might have been unable to adapt to the high SST in northern SCS. Specialist dinoflagellates in core A11–2 showed a rapid decline in RAB before generalists did. These specialists showed significant negative correlations with SST in core A11–2 (Fig. S6D) and had higher RAB in contemporary high-latitude surface sediments and surface waters (Fig. 4E and F), suggesting they are adapted better to lower SST than generalists. Additionally, in the stage II of Holocene in northern SCS, the relative sea levels in northern SCS continually increased until ~7.0 kyr BP [24]. The continually elevated sea levels caused TI reduction and further decreased the nutrient supply at core site A11–2. This is consistent with findings in a previous study that reductions in total dinoflagellate cyst concentrations and fluxes in northern SCS from ~10.4 to ~6.0 kyr BP were profoundly affected by reduced nutrient supply due to landward-migrated shorelines (i.e. TI reduction) [60]. Moreover, the decrease in Kuroshio intensity after 7.4 kyr BP further reduced nutrient supply at the site of core A11–2. Therefore, a combination of rising SST and decreasing nutrient supply caused the rapid decline in total dinoflagellates during the stage II.

In stage III, the RAB and SRI of dinoflagellates rose once more during this stage. Firstly, environmental factors such as SST, SSS, and TI were relatively stable in this stage, which may have promoted phytoplankton growth. Secondly, the increasing Kuroshio intensity likely enhanced nutrient supply, further promoting dinoflagellate growth. However, correlations between the paleoenvironmental factors and the RAB of dinoflagellates were weaker during Stage III than Stages I and II, suggesting that other factors have significantly influenced RAB and SRI of dinoflagellates during this stage. According to the archaeological site numbers and human population history, human activities in northern and southern China increased considerably during stage III (Fig. 2F–H). Similarly, based on pollen records, a previous study found a distinct shift from evergreen forest vegetation to Poaceae, Brassicaceae, Malvaceae, Rutaceae, and Anacardiaceae plants after 4.0 kyr BP in South Central China [61]. These plant types are associated with human cultivation practices, suggesting a significant increase in human activities after 4 kyr BP in south China. For instance, *Citrus grandis* (L.) Osbeck (Rutaceae) has >3000 years of cultivation history in China [62]. *Rhus verniciflua stokes* (Anacardiaceae), an economical tree, has been cultivated for >4000 years in China [63]. Cultivation of *Oryza sativa* (Poaceae) have been well developed in southern China after 4 kyr BP [64]. Moreover, researchers reconstructed the black carbon, clay mineral, and elemental records in a sedimentary core from the northwestern SCS and found a transition from mobile hunting-gathering to sedentary agricultural societies after 3 kyr BP [65]. Although the radiocarbon dating inherently has inaccurateness due to the sediment disturbance and contamination of allochthonous organic carbon, these various studies have found the rapidly increase of human activities in southern China after 3–4 kyr BP. Conceivably, deforestation, farming, and other anthropogenic activities due to population growth after 3–4 kyr BP potentially increased the nutrient input in north SCS and thus promoted RAB and SRI of dinoflagellates.

### Differential responses of generalist and specialist dinoflagellates to environmental and anthropogenic perturbation causing persistent increases in generalists

We found that with climate warming and environmental changes from early to late Holocene of northern SCS, the proportion of generalist dinoflagellate ASVs persistently increased (Fig. 4C and D). One of the possible reasons for the increase is that generalists are expected to adapt to a wider range of habitats and utilize a broader variety of resources than specialists [66]. For instance, dinoflagellates have three different trophic modes: autotrophic, mixotrophic, and heterotrophic modes. A previous study found that autotrophy-dominant mixotrophic dinoflagellates dominated global blooms more frequently than the heterotrophy-dominant mixotrophic dinoflagellates [67]. An important reason is the autotrophy-dominant mixotrophic dinoflagellates are able to feed on more diverse prey species than the heterotrophy-dominant mixotrophic dinoflagellates, allowing them to thrive in diverse conditions. Moreover, some dinoflagellates are eurythermal species, whereas some others are stenothermal species. For example, the laboratory experiments showed that *P*. *shikokuense* was able to grow at temperatures ranging from 10 to 27°C [68], whereas the optimum temperature range of *C. fusus* was narrower (26–28°C) [11]. As a result, the wider adaptation allows generalists to have a stronger ability to tolerate environmental disturbances compared to specialists [41, 66]. During the Holocene in the northern SCS, climate and environmental factors changed rapidly (e.g. the increase of mean annual SST and the increase of human activities after 4 kyr BP), whereas the RAB of generalist ASVs remained stable, that of specialist ASVs was highly dynamical (Fig. S6C). As a result, generalists were favored in the Holocene in the northern SCS due to rapidly changing climate and environmental factors.

The increased human activities in south China after 4 kyr BP might have caused marine habitat loss and fragmentation, leading to a decline in some specialist dinoflagellates. For example, eutrophication induced by human activities promotes the loss of seagrass meadows, seaweeds, coral reefs, fish, and low-nutrient habitats, and thus leads to a decline in specialists [69]. Previous studies have found this trend in various organisms and showed that eutrophication is unfavorable to specialists which led to homogenization of microbial and cyanobacterial communities in lakes [17, 70] and homogenization of copepod communities in Mediterranean Sea [71] by increase of generalists. Based on niche modeling and > 540 000 global phytoplankton observations, researchers have also found phytoplankton generalists perform better in variable and nutrient-rich environments than specialists [72].

Generalists are assumed to have stronger dispersal ability than the specialists [73]. The stronger dispersal ability for generalists may further promote the domination of generalists. Generalists benefit from their higher environmental adaptation, which allows them to disperse gradually to neighboring habitats. In contrast, specialist species as their narrow environmental adaptation creates a barrier that prevents them from reaching other favorable habitats. Therefore, when the environmental conditions change in a habitat, specialists from other remote habitats that adapt to these changes may be unable to reach there whereas some generalists can, leading to the domination of generalists.

### Ecological implications of increasing generalist dinoflagellates: potential expansion rather than simply poleward migration of dinoflagellate HABs under global change

In contemporary oceans, the disturbance and loss of marine habitats as well as climate warming has increased at unprecedented rates after the first Industrial Revolution [69]. Habitat disturbances and climate warming may lead to intense competition between specialists and generalists, as well as extinction of specialists due to their low adaptation to changing abiotic and biotic conditions. The loss of specialist dinoflagellates and the increasing dominance of generalist dinoflagellates may have some significant negative effects on marine ecosystems.

One of the main negative effects for increasing dominance of generalist dinoflagellates is that dinoflagellate blooms of major generalists may expand geographically rather than simply migrate to higher latitudes under climate warming and environmental changes, due to the adaptation of generalists to wider niches. We found that the most typical generalist genera (e.g. *Noctiluca*, *Gymnodinium*, and *Prorocentrum*) in core A11–2 (Fig. 5F, the asterisk) were also taxa that have contributed to the most frequent bloom records from south to north of China (Fig. 6C–F, the asterisk). For example, *Gymnodinium* and *Noctiluca* were the most typical and abundant generalist genera in core A11–2 (Fig. 5F). Also, *Gymnodinium* and *Noctiluca* dominated blooms in both north (Fig. 6C) and south China (Fig. 6F), which spans 22° in latitude. Moreover, the typical and abundant generalist genera (*Prorocentrum*) in core A11–2 also frequently bloomed in coastal regions of south China from SH to Hainan (HN) (Fig. 6D–F). The typical bloom-forming dinoflagellate species in Chinese coastal regions such as *Gymnodinium catenatum* (16 to 28°C) [74], *N. scintillans* (14 to 28°C) [75], and *P*. *shikokuense* (10 to 27°C) [68] displayed high growth rates under widely varying temperature conditions. Moreover, global eutrophication may further expand the distribution areas of generalist dinoflagellates. Eutrophication provides abundant nutrient resources, facilitating the global expansion of dinoflagellate blooms. Moreover, eutrophication may lead to loss of some specific habitats, and thus favors the generalists.

Our results further showed that an increase in generalists and a decrease in specialists led to homogenization of dinoflagellate communities (Fig. 7), which negatively affected the co-occurrence of marine planktons. Dinoflagellates have multiple interactions with other marine planktons and animals such as predator–prey, parasite–host, cooperation, and competition. For example, in marine food web, dinoflagellates is an important primary producer, and are also known to feed on diverse types of prey, including bacteria, diatoms, other dinoflagellates, and other picoeukaryotes [76]. In turn, dinoflagellates serve excellent prey for mixotrophic and heterotrophic protists as well as metazoa [76]. Moreover, dinoflagellate is an important marine parasite (e.g. infect other dinoflagellates and ciliates) and parasitic host, as well as has symbiotic relationships with other algae, protozoa, and metazoa [77]. Our analysis of co-occurrence networks showed that specialist dinoflagellate ASVs are linked to more diverse microbial eukaryotic ASVs (such as Fungi, Chlorophyta, Bacillariophyta, Cercozoa, Ciliophora, Perkinsea, Pseudofungi, and Chrysophyceae) than generalists. This indicates that the loss of specialists may simplify the co-occurrence network of marine planktons, i.e. plankton potential interactions (Fig. 8), and thus decrease the stability of the co-occurrence network due to processes including decrease in selectivity of cooperator and prey, and decrease in resistance to disturbance [78, 79]. Potential outcomes of such functional biodiversity include decreases in resource use efficiency, productivity (e.g. fisheries), stability, and other ecosystem services [80–83].

## Conclusions

This study demonstrates that sedaDNA analysis allows a comprehensive reconstruction of the response of dinoflagellate assemblages to climate and environmental changes in the Holocene in northern SCS, providing compatible results with conventional paleogeography (based on dinosterol content). We found that with the warming and environmental changes in the early and middle Holocene in northern SCS, RAB and SRI of dinoflagellates firstly increased (~12 to ~9.1 kyr BP) and then decreased to the lowest value (~9.1 to ~4.3 kyr BP). However, RAB and SRI of dinoflagellates increased again after ~4.3 kyr BP, which was closely related to the increase in human activities.

The environmental and climate changes during the Holocene in northern SCS caused the generalist dinoflagellates to gradually dominate the total dinoflagellate community. These generalists were also abundant throughout contemporary low and middle latitude regions of global oceans although specialists were more abundant at higher latitudes. Particularly, the dominated generalist genus in core sediment such as *Gymnodinium*, *Prorocentrum*, and *Noctiluca* also frequently bloomed across the entire Chinese coastal regions from south to north in recent 40-years. Due to adaptation to a wider range of habitats, dinoflagellate blooms caused by generalists may show expansion rather than simply migration patterns toward higher latitudes under global warming scenarios. Moreover, domination of generalists resulted in homogenization of dinoflagellate communities, which could have significant effects on co-occurrence of marine plankton.

## Supplementary Material

3_Supplementary_Figures_wraf095

4_Supplementary_Tables_wraf095

## Data Availability

The raw sequence data from sediment samples have been deposited in the Genome Sequence Archive (National Genomics Data Center, China National Center for Bioinformation / Beijing Institute of Genomics, Chinese Academy of Sciences) (https://ngdc.cncb.ac.cn/gsa/) with the accession number CRA007427, CRA018265, and CRA018173.
