# Supplementary material for: Holocene and contemporary marine dinoflagellate community patterns predict expansion of generalist dinoflagellate blooms in warming oceans
Source: ISME J. 2025 May 14;19(1):wraf095. doi: 10.1093/ismejo/wraf095 (PMC12133096; doi:10.1093/ismejo/wraf095)
Supplement: 3_Supplementary_Figures_wraf095 [file 3_supplementary_figures_wraf095.pdf]

Supplementary Information

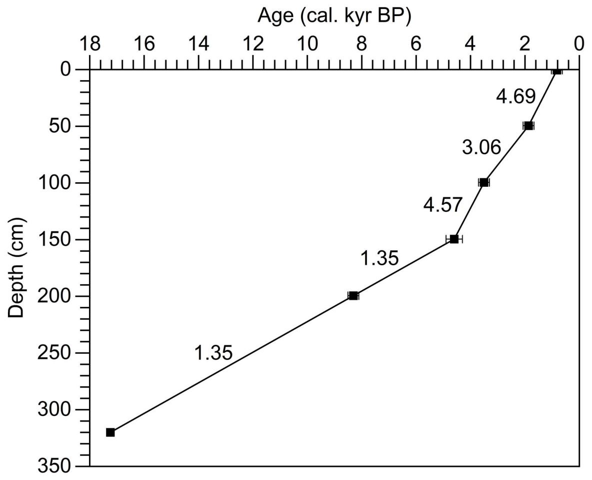

Figure S1 Age-depth relationship of core A11-2 shown by the calibrated dates plotted against sediment depth. Numbers are the sedimentation rates (cm kyr^{-1}) calculated by linear interpolation between two adjacent ^{14}C ages.

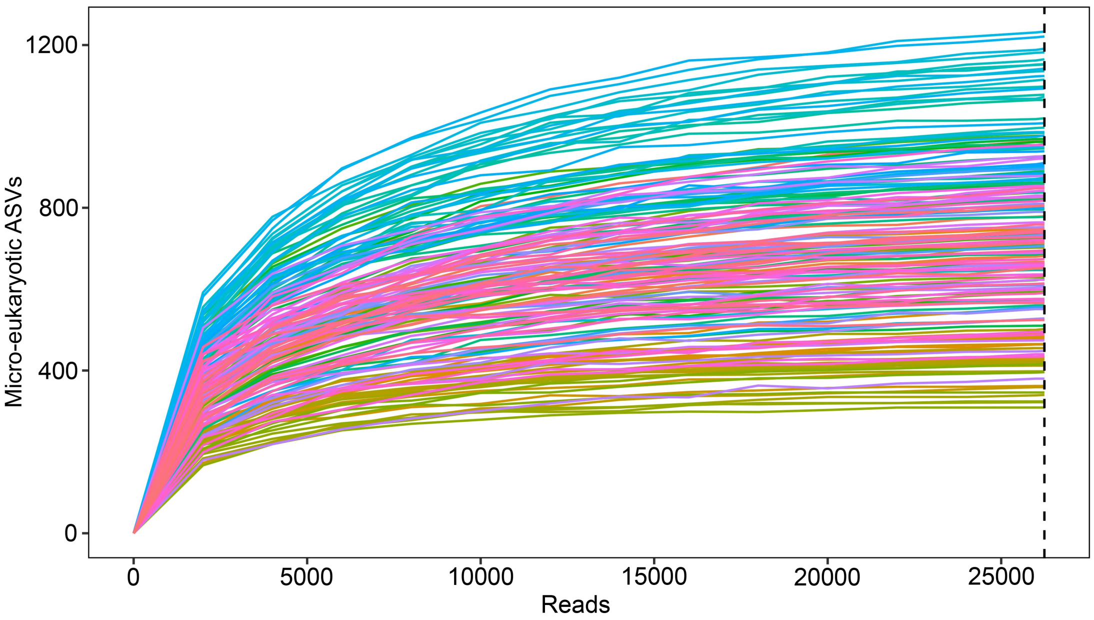

Figure S2 The rarefaction curves for 171 samples in core A11-2 according to micro-eukaryotic 18S rRNA gene amplicon sequencing.

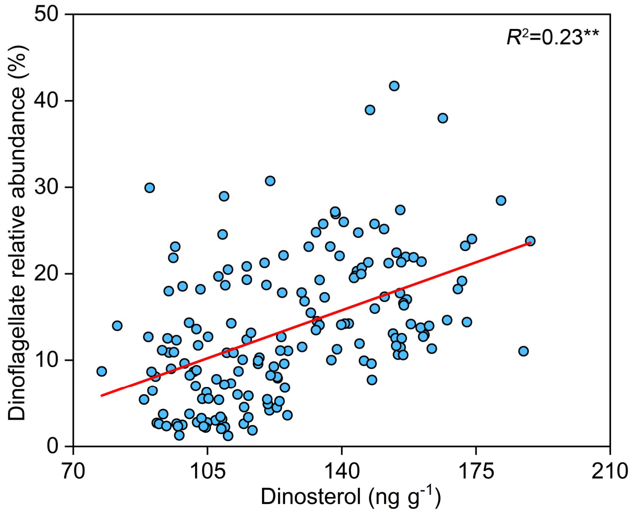

Figure S3 Data scatter and linear regression showing the relationships between biomarker of dinoflagellate absolute abundance (dinosterol) and dinoflagellate relative abundance (% dinoflagellate sequences over total micro-eukaryotic sequences, RAB) of sedaDNA in core A11-2.

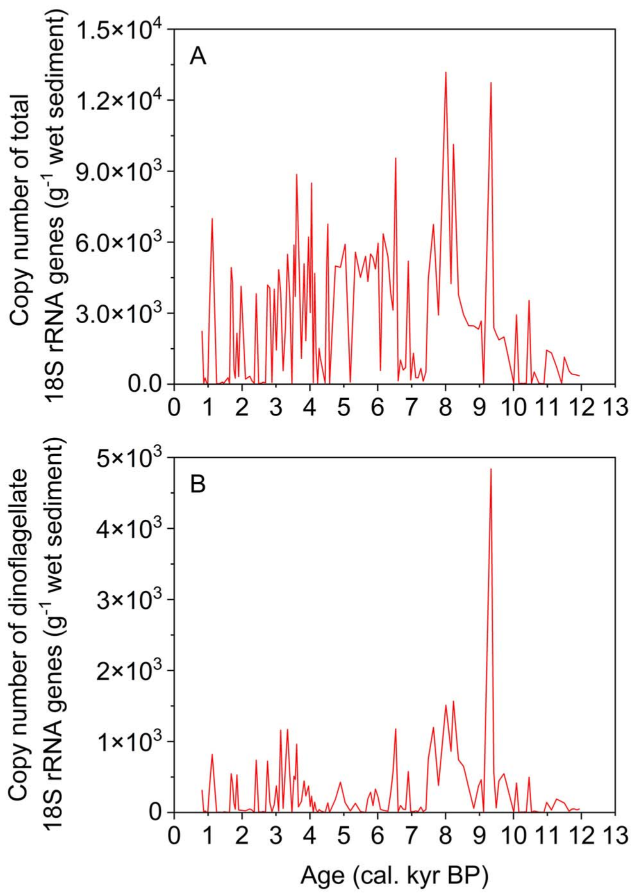

Figure S4 Variations in total 18S rRNA gene copy number (A) and dinoflagellate 18S rRNA gene copy number (total 18S rRNA gene copy number × dinoflagellate RAB) (B) in core A11-2 during the Holocene.

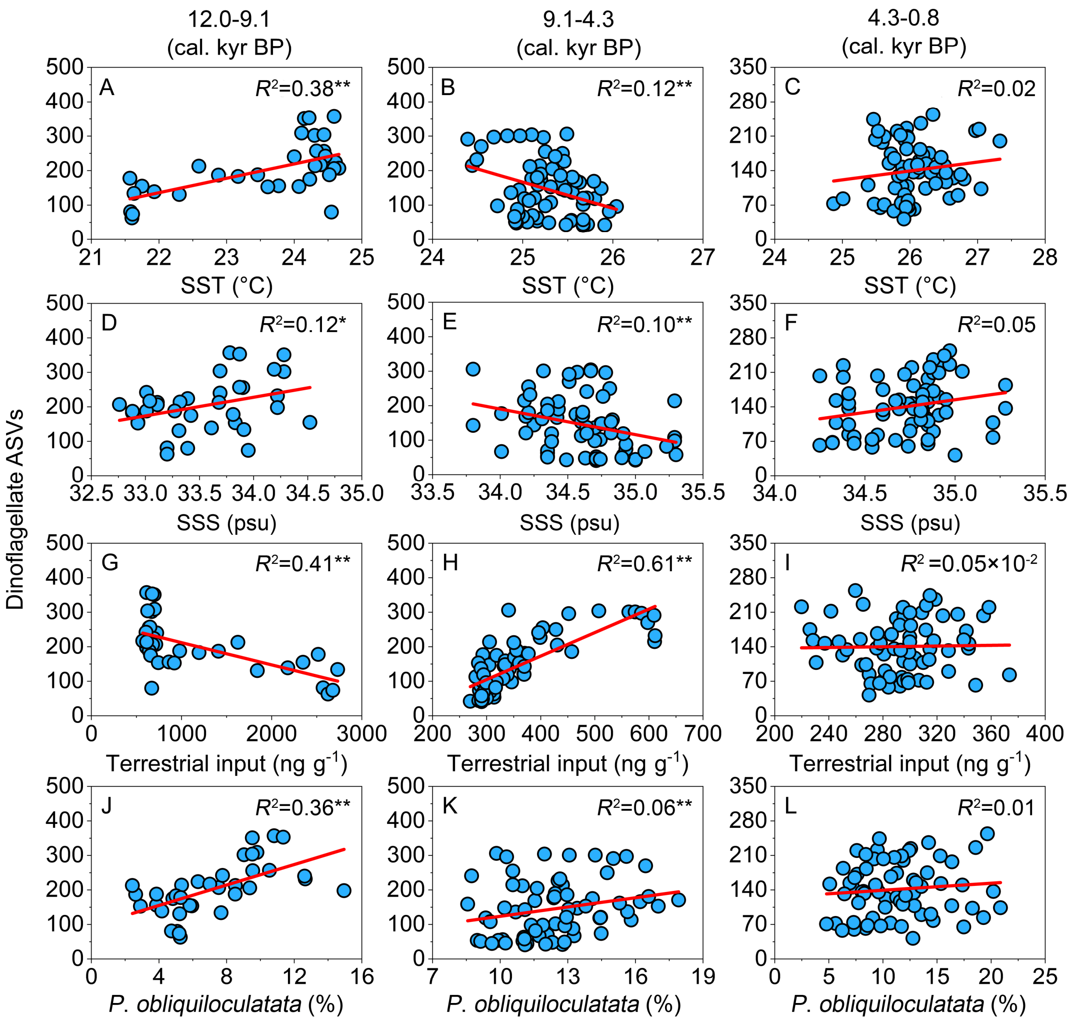

Figure S5 Data scatter and linear regression showing the relationships between dinoflagellate ASV number in core A11-2 and palaeoenvironmental factors during the period from ~12 to ~9.1 kyr BP (A, D, G, J), from ~9.1 to ~4.3 kyr BP (B, E, H, K) and from ~4.3 to 0.8 kyr BP (C, F, I, L). * - $P <$ 0.05, ** - P <0.01. *P. obliquiloculatata,* the formaminifera *Pulleniatina obliquiloculata.*

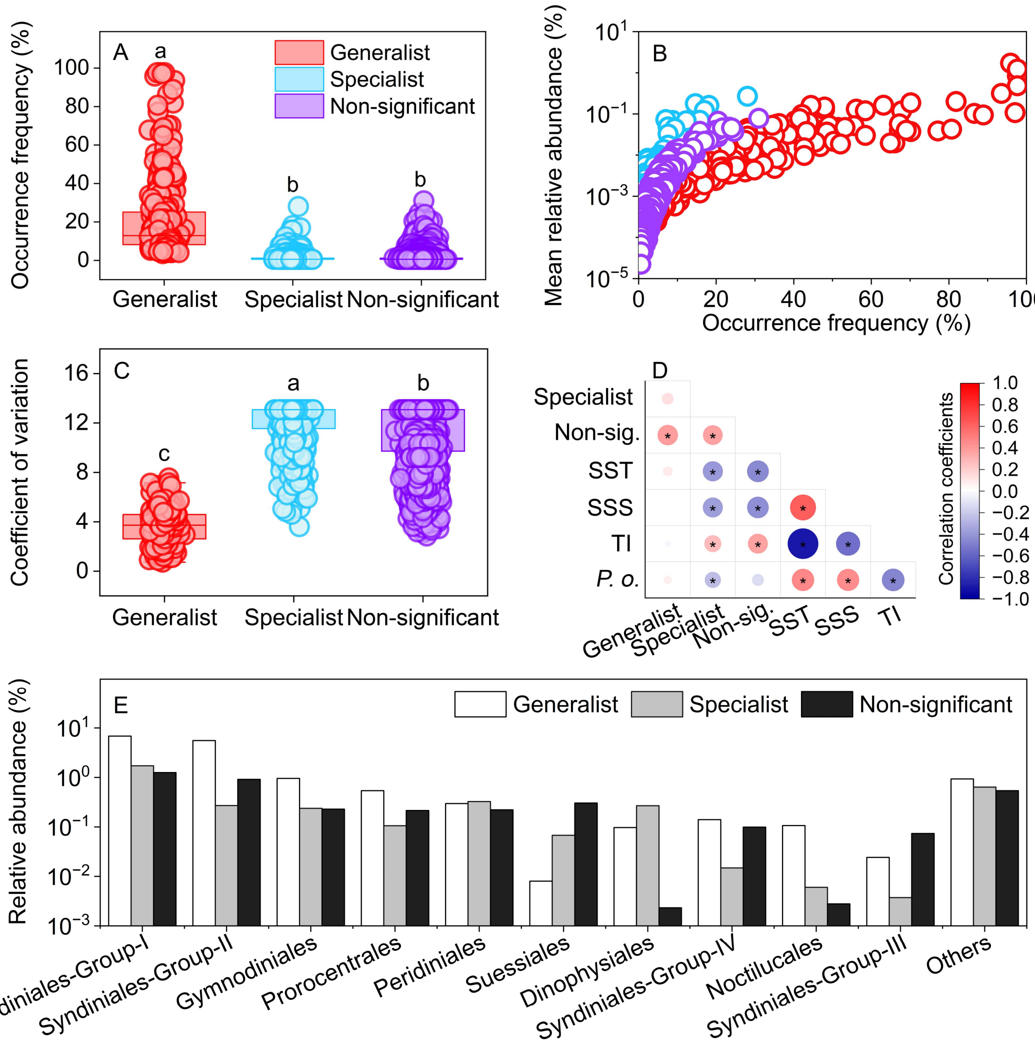

Figure S6 Occurrence frequency of generalist, specialist and non-significant dinoflagellate ASVs (A) and their correlations with their mean RAB (B) in core A11-2. Variation coefficients of generalist, specialist and non-significant dinoflagellate ASVs in core A11-2 (C). Correlations between dinoflagellate RAB and palaeoenvironmental factors in core A11-2 (D, * - $P < 0.05$). Composition of family level of dinoflagellate ASVs in core A11-2 (E).

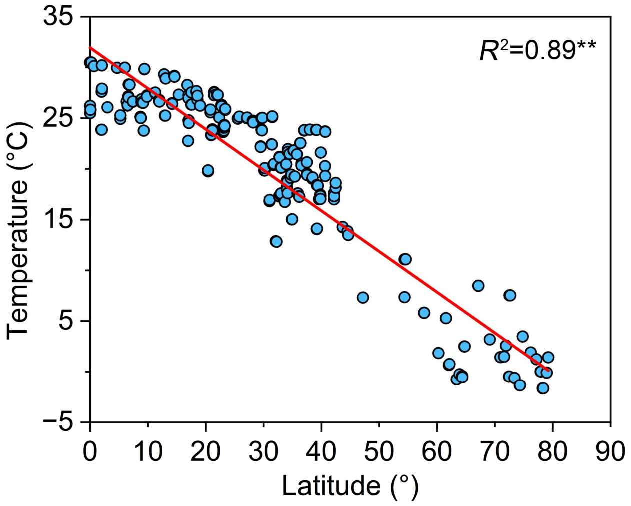

Figure S7 Data scatter and linear correlation between sea surface temperature (SST) and latitude in contemporary water samples.
